# Dietary intake temporal patterns during early childhood in relation to diet quality in middle childhood and the role of family characteristics: the GECKO Drenthe cohort

**DOI:** 10.1007/s00394-026-03934-8

**Published:** 2026-03-09

**Authors:** Jie Yang, Gerjan Navis, Monica Mars, Eva Corpeleijn

**Affiliations:** 1 Department of Epidemiology, University of Groningen, University Medical Centre Groningen, Postbus 30.001, 9700 RB Groningen, The Netherlands; 2 Department of Internal Medicine, Division of Nephrology, University of Groningen, University Medical Centre Groningen, Groningen, The Netherlands; 3 Division of Human Nutrition and Health, Wageningen University, Wageningen, The Netherlands

**Keywords:** Dietary intake temporal patterns, Dietary consistency, Infancy, Siblings, Socio-economic status, Jarred baby foods

## Abstract

**Purpose:**

Early childhood food intakes may influence subsequent diet quality. This study described dietary intake temporal patterns of specific food groups from infancy to 3 years. It explored associations with diet quality at 10/11 years and investigated how family characteristics affect these dietary patterns.

**Methods:**

Food group intake was assessed in 2552 children (49.9% boys) at several time points from 7 months to 3 years (GECKO Drenthe cohort). Latent class analyses (LCA) identified temporal patterns for healthy foods (fruit, vegetable, bread type, and dairy) and unhealthy foods (‘meat and fish’, convenience meals, savory snacks, sweet snacks, and sugar-sweetened beverages [SSB]). Associations with diet quality at 10/11 years (n = 856) were assessed using linear regression. Associations with family characteristics (n = 2256) were examined using multinomial logistic regression.

**Results:**

Three distinct temporal patterns were identified for each food group, differing in intake levels and changing over time. Dietary intake patterns were associated with intake of the same food group at 10/11 years (*p* < 0.05). The weakest tracking was observed for ‘meat and fish’, while the strongest tracking was seen for SSB. Higher intake patterns of healthy foods were associated with better diet quality at 10/11 years, while the opposite was true for unhealthy foods. Unhealthier food intake patterns were related to lower parental education and household income, younger parental age, higher parental BMI, smoking during pregnancy, and having siblings at birth.

**Conclusion:**

Food group intakes from infancy to age 3 were associated with subsequent diet quality. Promoting healthy eating in early life is essential for long-term dietary health.

**Supplementary Information:**

The online version contains supplementary material available at 10.1007/s00394-026-03934-8.

## Introduction

Diets in childhood are not only important for the growth and development of children [1]; they may also establish enduring dietary temporal patterns that influence diet quality [2, 3]. Poor diet quality affects both the health of children and adults, leading to an increased risk of noncommunicable diseases, such as obesity [4], cardiovascular disease [5], and diabetes [6]. In 2017, poor diet was associated with an estimated 10.9 million deaths and 255 million disability-adjusted life years (DALYs) globally, making it one of the most significant metabolic and behavioral risk factors influencing the global burden of disease [7]. Therefore, adopting healthy eating habits at an early age is essential and can provide substantial benefits for the entire population.

Repeated dietary assessments are essential for identifying temporal patterns of dietary intake and evaluating the cumulative impact of diet on health outcomes. This longitudinal approach provides a framework to understand how diets evolve, captures broader dietary shifts, particularly as populations experience epidemiological and nutritional transitions that change the food environment [8]. Previous studies suggested that the development of diet may have occurred primarily before the age of 3 years [9]. Indeed, the transition from a milk-based infant diet to a fully diverse toddler diet may be an ideal time to ensure children initiate healthy dietary habits. However, dietary tracking has been studied in older children [3, 10, 11], some studies have assessed overall diet quality [2, 9], or have compared dietary behaviors at two time points [12, 13]. Few studies have explored detailed food groups intake tracking beginning in infancy to determine whether healthy dietary habits in early life are sustained, or deteriorate with the increasing autonomy of the child. The Melbourne Infant Feeding Activity and Nutrition Trial Program (n = 467) revealed consistent tracking of intakes of three food groups (fruit, vegetable, discretionary foods) from 9 months to 5 years, representing the only study to date that has monitored food group intake starting from infancy [14]. The Avon Longitudinal Study of Parents and Children compared food groups intakes at 18 months to those in the same children at 3.5 years and found an increase in the consumption of discretionary foods between the 2 ages [15]. Two longitudinal studies covering slightly older ages, 2 to 5 years [13], and 3 to 9 years [11], showed that the dietary patterns found were relatively consistent over time. Consequently, a significant gap exists in understanding the development of food-group-specific dietary patterns from infancy through mid-childhood. To address this, our study provides longitudinal data on nine food groups and four subgroups across three to seven time points, from infancy to 3 years of age offering highly reliable insights into dietary tracking and its progression over time to inform targeted interventions.

Parental concerns regarding the long-term health impacts of commercial baby foods remain prevalent [16]. Current research underscores the importance of promoting a wide range of flavors and textures in both fresh and commercial baby foods to meet the nutritional and developmental needs of infants and young children [17]. However, limited evidence is available on how early exposure to commercial baby foods, as opposed to fresh foods, may influence diet quality in later childhood. Further investigation is needed to clarify the role of these foods in shaping early dietary patterns and their implications for child health.

While others used group-based trajectory modeling to track diet quality trajectories [2, 9], and principal component analysis to identify cross-sectional dietary patterns (e.g. “healthy” pattern) [11, 13], we propose repeated-measures latent class analysis (LCA) to assess temporal patterns of dietary intake across multiple food groups. A key advantage of LCA in this context is its capacity to model dynamic changes within multiple food groups and pinpoint critical time points where dietary trends shift. This person-centered approach allows us to classify individuals into homogeneous latent classes based on their entire longitudinal response profiles [18]. It provides an estimate of the smallest number of homogeneous groups (latent classes) that best represent the population. In this way, the derived classes represent a group of individuals who are homogeneous in food group intake and their changes within, but heterogeneous across classes.

In addition to understanding (the change in) diets over time, it is critical to assess how these temporal patterns can differ among different groups of people in society, as this knowledge is essential for identifying groups at risk and designing targeted interventions. For example, understanding how socioeconomic status (SES) is linked to children’s diets is important for tackling SES inequalities [19]. Existing evidence suggests that parental educational level may influence the home food environment through nutritional knowledge and food-related parenting practices [20]. Additionally, low-income individuals may experience resource constraints and are more likely to reside in neighborhoods with limited access to healthy food options, leading to poor access to foods [21]. While previous studies have demonstrated positive associations between parental education, income, and healthy dietary patterns in children and adolescents [13, 22–24], the factors linked to unhealthy diets remain inconsistent [13, 22, 25, 26]. These mixed findings highlight the need for further research to investigate how family characteristics affect children’s dietary intake patterns.

The present study had three aims: First, to describe the longitudinal dietary intake temporal patterns of different food groups from early childhood, up to 3 years of age, in the GECKO (Groningen Expert Centre for Kids with Obesity) Drenthe birth cohort. Second, to explore how the dietary intake temporal patterns of healthy or unhealthy food groups in early childhood are related to diet quality in middle childhood at 10/11 years of age. And third, to evaluate how SES and family characteristics are related to children’s dietary intake temporal patterns. Previous studies have demonstrated the tracking of dietary habits throughout childhood, suggesting that poor dietary patterns established at a young age may predict the continuation of unhealthy eating behaviors later in life [2, 3, 13, 14]. Therefore, we hypothesized that children who exhibit unhealthy dietary intake temporal patterns during early childhood are more likely to maintain poor diet quality by age 10/11 years, with their dietary temporal patterns potentially influenced by family characteristics.

## Methods

### Study population

The GECKO Drenthe birth cohort is a Dutch population-based cohort comprising around 3000 children born between April 2006 and April 2007. It is set up to study the determinants and development of children’s diet and childhood overweight and obesity. The design and methodology of the GECKO Drenthe birth cohort have been described elsewhere [27]. In short, all pregnant women in Drenthe with an expected delivery date between April 2006 and April 2007 were invited to participate in this study by their midwives, general practitioners, or gynecologists. After informed consent was obtained, these women were included during the third trimester of their pregnancy or within 6 months after delivery. The GECKO Drenthe birth cohort was conducted in accordance with the Declaration of Helsinki and was approved by the Medical Ethics Committee of the University Medical Centre Groningen, the Netherlands (Medical ethical approval ID: 2005.260). Informed consent of participation was given by parents or guardians. The cohort is registered at www.birthcohorts.net (ID: 138).

Out of 2684 children with valid dietary data (after correcting decimal default errors due to computer processing), 2552 were included in the study to monitor food intake; these children had more than two food intake assessments between 7 months and 3 years of age. Of these, 856 had dietary data at ages 10/11 years and were included in the analysis examining food intake and diet quality in middle childhood (see supplementary Fig. S1 for a flow chart). The number of children included in the dietary intake pattern analyses varied across food groups due to differences in the age at which specific foods were typically assessed (e.g., fruits and vegetables from 7 months, bread from 14 months, etc.). As a result, sample sizes varied by food group (supplementary Fig. S1).

### Data collection

#### Anthropometric measurements

The weight and height of the children were measured during routine health examinations conducted by trained staff of the Youth Health Care centers (YHC) at ages 3 and 10/11 years. Body weight was assessed using an electronic scale and rounded to the nearest 0.1 kg. Height was measured in the standing position against a wall and rounded to the nearest 0.1 cm.

#### Dietary data

We assessed the diets after the introduction of solid foods, which in the Netherlands is between 4 and 6 months of age. A total of 8 visits were conducted throughout the study; at the age of 7, 9, 11, 14, 18, and 24 months, 3 and 10/11 years. At ages 3 and 10/11 years, a validated food frequency questionnaire (FFQ) comprising 71 food items, specifically designed for Dutch children, was filled out by their parents [28]. Seven response categories ranging from ‘not used’ to ‘6–7 days a week’ were applied to assess the frequency of food consumption. Portion sizes were assessed with fixed unit portion sizes (e.g. slices of bread) or common household measurements (e.g. cups and spoons). Data were processed with the help of standardized portion sizes to calculate the total intake of each food item in grams per day. Subsequently, daily energy and nutrient intakes were calculated using the Dutch Food Composition Database 2011 [29]. For each food item, energy intake was calculated by multiplying the estimated daily intake (g/day) by its corresponding energy value (kcal per 100 g), and the total daily energy intake was obtained by summing the energy contributions of all food items. From the FFQ, data were extracted on the consumption of specific food groups, including fruit, vegetable, bread, dairy, meat and fish, convenience meals (including pizza, pancakes, and patat—French fries in Dutch—this group of foods indicates typical convenience meals for Dutch children), savory snacks, sweet snacks, and sugar-sweetened beverages (SSB).

Possible under or overreporting with the FFQ was excluded by the use of the Goldberg cutoff method, based on the ratio of the reported energy intake and the basal metabolic rate [30], calculated with the Schofield equation [31]. As a result, 58 children with an energy intake/baseline metabolic rate ratio below 0.87 (n = 55) or above 2.75 (n = 3). Moreover, 4 children with incomplete FFQ data were excluded from the final analysis.

In the younger ages, 7, 9, 11, 14, 18, and 24 months, the consumption of specific food groups was evaluated using a series of self-administered food intake questionnaires; questioning age-appropriate foods. These questionnaires contained questions about 1) the type and amount of fruit and vegetable (from 7 months onward), 2) the main type of bread consumed: white, brown (made from a mixture of refined flour and whole meal flour), or wholegrain (from 14 months onward),3) the amount of dairy (from 14 months onward), 4) the amount of meat and fish (from 18 months onward), 5)the frequency of convenience meals (from 18 months onward), savory snacks (from 18 months onward), sweet snacks (from 14 months onward), and SSB (from 18 months onward). More detailed information about these food intake questionnaires is presented in supplementary Fig. S2 and Table S1.

### Development of dietary intake temporal patterns

The information reported in the food intake questionnaires from 7 months to 3 years was used to track the trends of diet across several food groups: fruit, vegetable, bread type (white, brown, wholegrain), dairy, and a combined ‘meat and fish’ category (due to meat and fish being assessed together in a single question and the relatively low consumption of fish at this age [32]), as well as convenience meals, savory snacks, sweet snacks, and SSB. For fruit and vegetable, jarred and fresh forms were analyzed separately, reflecting common Dutch practices of initiating complementary feeding with ready-to-eat jarred products. Dietary intake temporal patterns were established separately for each food groups. For bread, patterns were defined by the predominant type consumed, whereas for other food groups, patterns were based on food intake level (g/day), categorized as low, medium, or high. The cut-offs for each food group are presented in supplementary Table S2. The cut-offs were divided according to frequency and relevance. Taking fruit, for example, one jar of fruit is equivalent to 194 g and one piece of fresh fruit is equivalent to 120 g. To standardize one portion of fresh and jarred fruit within the same food group, we classified fruit consumption into three categories: < 100 g as low, 101–200 g as medium, and > 200 g as high.

#### Diet quality indices

Based on the dietary data obtained with the FFQ at age 10/11 years, three different types of diet quality indices were calculated: the lifelines diet score (LLDS), the dietary approaches to stopping hypertension (DASH) score, and the Mediterranean diet score (MDS), all representing a different dimension of diet quality.

The LLDS, developed based on international evidence on diet-disease relationships outlined in the 2015 Dutch Dietary Guidelines, serves as a reliable indicator of Dutch dietary habits [33]. Methods for calculating this diet score have previously been developed and described [33]. LLDS includes eight health-positive food groups (vegetable, fruit, whole grain products, legumes and nuts, fish, oils and soft margarine, unsweetened dairy, and tea), and three health-negative food groups (red and processed meat, butter and hard margarine, and SSB). Daily consumption in g/1000 kcal of each food group is divided into quintiles, scored on a scale of 0–4 (reverse for health-negative food groups), and summed, with higher scores indicating better diet quality.

The DASH diet is specifically designed to maintain healthy blood pressure [34]. Recent research involving a sample of 425 Iranian children aged 6 to 18 years [35] and 1570 Canadian children with a mean age of 12.4 years [36] suggests that adherence to the DASH diet may play a substantial role in the prevention of overweight in children. To calculate the DASH diet score, five food groups (vegetable, fruit, whole grain products, legumes, and nuts; and low-fat dairy) with positive health effects, and three food groups (sodium, red and processed meat, and SSB) with negative health effects were identified. All intakes were adjusted for energy. Each food group is divided into quintiles based on intake, scoring 0–4 (the opposite for negative groups) and summed, with higher scores indicating a better diet quality. The details to calculate this diet score have been previously described [37].

The MDS assesses adherence to the traditional Mediterranean diet, which has demonstrated protective effects on the incidence of cardiovascular diseases [38]. Studies have shown that compliance with the Mediterranean diet is associated with the prevention and control of childhood obesity in the pediatric population [39]. In the MDS, fruit, nuts, vegetable, legumes, grains, potatoes, and fish are classified as beneficial components of the Mediterranean diet, while meat and dairy products are classified as detrimental components. All intakes were adjusted for energy. A score of 0 or 1 point is assigned according to the median intake of beneficial or detrimental components, respectively. Except for fish intake, children who did not consume fish are assigned a value of 0 and those who did are assigned a value of 1. Fat intake is scored based on the ratio of monounsaturated to saturated lipids. The final MDS score ranged from 0 to 9 points, with a higher score indicating better diet quality. The details of this score have previously been described [40].

### Family characteristics

During the pregnancy period, comprehensive demographic and family characteristics were collected through a detailed questionnaire completed by both parents. Furthermore, data were supplemented by midwives, gynecologists, and clinical records. The variables used for this analysis included maternal and paternal education level, age at birth of the child, occupational status, smoking habits during pregnancy, pre-pregnancy height and weight, household income, and the presence of siblings at birth. Maternal and paternal characteristics were analyzed separately. Education levels were divided into three categories: low (no education, primary school, lower vocational or lower general secondary education), middle (intermediate vocational training or higher secondary education), and high (higher vocational or university education). Parental age at birth of the child was categorized into four groups: 16–25 years, 26–30 years, 31–35 years, and 36 years and older. Occupational status was dichotomized as employed (employed, or self-employed) or unemployed (unemployed, student, domestic tasks, inactive, or other receiving benefits or pension). Pre-pregnancy weight status was classified according to Body Mass Index (BMI) as follows: not overweight (BMI < 25 kg/m^2^), overweight (25 ≤ BMI < 30 kg/m^2^), and obesity (BMI ≥ 30 kg/m^2^). Smoking behavior during pregnancy and the presence of siblings were dichotomized as: yes or no. Household income in this study was defined by the “Equivalized Household Income Indicator (EHII),” a standardized, cross-cohort comparable income indicator developed by Pizzi et al*.* that provides a direct measure of material resources [41]. Based on tertiles of estimated household income, income levels were categorized into three groups: low (< €1752/month), middle (€1752–2216/month), and high (> €2216/month).

### Statistical analysis

To describe the temporal patterns of dietary intake of the different food groups, LCA was applied to the food intake levels from 7 months to 3 years of age to identify unique patterns. We evaluated latent class models with 2 to 5 classes to determine the optimal number of classes. The optimal number of classes was determined by evaluating the fit, interpretability, parsimony, and stability/identification of the model. The model fit indices, including the Akaike information criterion (AIC) values, the Bayesian information criterion (BIC) values, and the entropy. The analysis relied on a variety of fit criteria, of which the BIC has been shown in simulations to perform particularly well in selecting the ‘correct’ latent class model [42]. Each child was assigned to the class corresponding to their highest posterior probability. Classification uncertainty inherent to probabilistic assignment was acknowledged.

To explore if the dietary intake temporal patterns of healthy or unhealthy food groups in early childhood predict food intake in middle childhood, we employed chi-square tests for categorical variables, non-parametric Kruskal–Wallis test and Mann–Whitney U test for continuous variables with a skewed distribution. Next, linear regression analysis was conducted to examine the association between dietary intake temporal patterns and diet quality, measured by the LLDS, the DASH score, and the MDS. In this analysis, potential child-related confounders (child’s sex and age) were added to the model. To determine to what extent family characteristics explained the association between dietary intake temporal patterns and later diet quality, maternal education level, maternal smoking during pregnancy, and the presence of siblings at birth were included in the model. 17 children with missing data for at least one covariate were excluded from the analysis.

To evaluate how SES and family characteristics are related to children’s dietary intake temporal patterns, multinominal logistic regression analysis was employed to explore the associations between socioeconomic and parental factors and dietary intake temporal patterns in early childhood. A backward stepwise approach was used to select significant factors. Variables were chosen based on existing literature [43–45] as anticipated predictors of dietary intake temporal patterns or exposures, including parental education level, occupational status, weight status, smoking status during pregnancy, age at birth, household income, and the presence of siblings at birth. Due to the high correlation between maternal and paternal characteristics, these factors were analyzed separately to investigate their contributions to dietary intake temporal patterns.

For a sensitivity analysis, linear regression analysis was repeated using recalculated diet quality scores that excluded the component corresponding to the food groups of interest. This applied to fruit, vegetable, dairy, meat and fish, and SSB, as these were the food groups included as components of the diet quality score. For example, when examining associations between temporal patterns of fruit intake and overall diet quality, the fruit component was omitted from the score. This approach allowed us to investigate the independent contribution of specific dietary intake temporal patterns to overall diet quality, while minimizing potential inflation from the intake of the group under investigation.

Children with missing values in multiple covariates were excluded from the regression analysis. Assumptions for regression analysis were verified and collinearity between variables was assessed. Data analysis was performed with IBM SPSS Statistics version 28 and R version 4.4.1. Statistical significance was set at *p* < 0.05.

## Results

### Participants characteristics

Supplementary Table S3 illustrates the basic and family characteristics of included participants. Of the participating children included in the food intake patterns, 49.9% were boys. Most parents had a medium or high level of education. Employment was prevalent; with 90.3% of mothers and 97.8% of fathers in the workforce. The median household income was €1946 (P10-P90: €1466-€2561). A total of 14.1% of mothers and 35.0% of fathers reported smoking during the pregnancy. Furthermore, 59.6% of the children had one or more siblings at birth.

### Dietary intake temporal patterns in early childhood

**Fig. 1:**
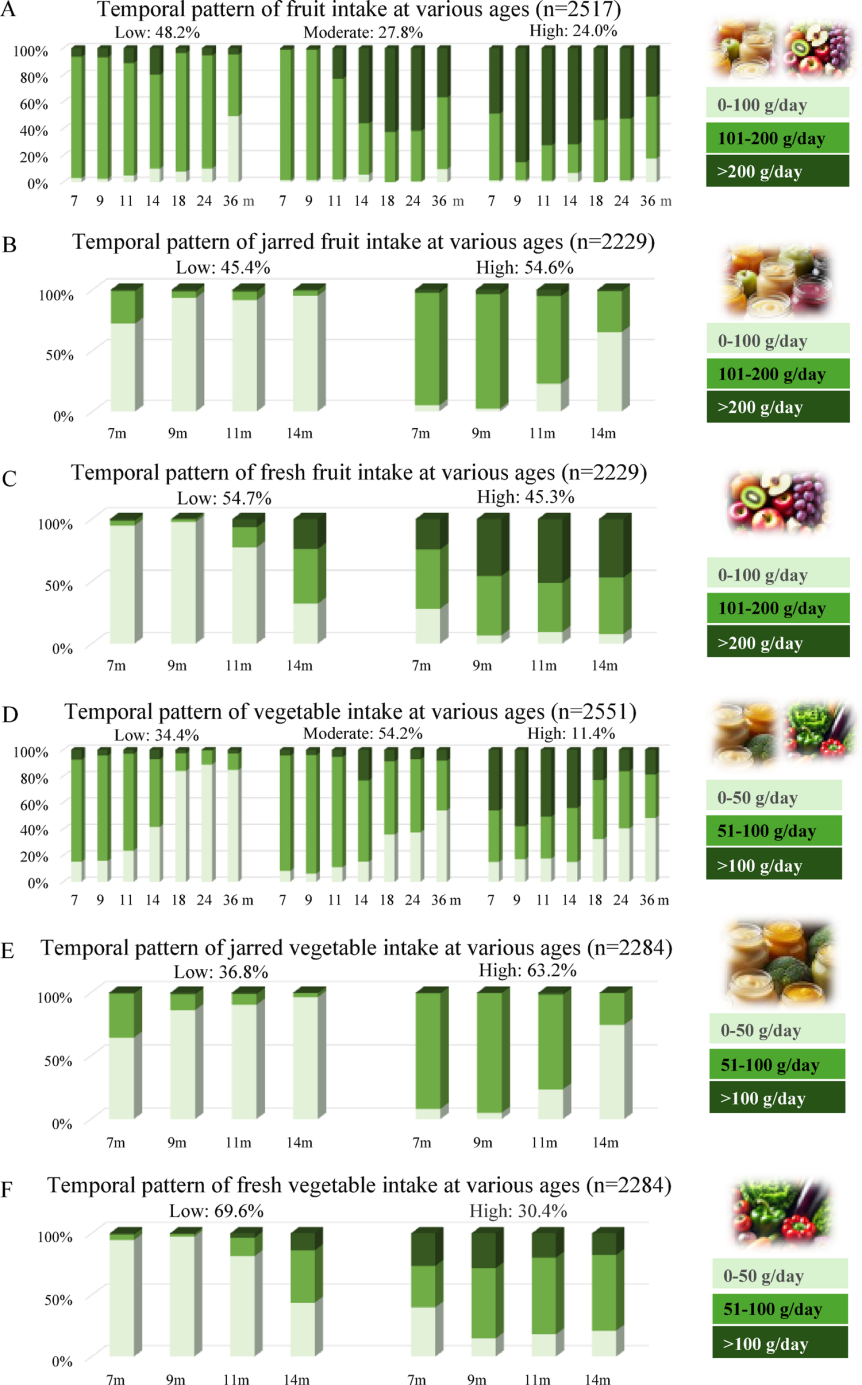
Dietary intake temporal patterns for fruit and vegetables showing the percentage of children with low, medium, or high intakes at each age from ages 7 months to 3 years

**Fig. 2:**
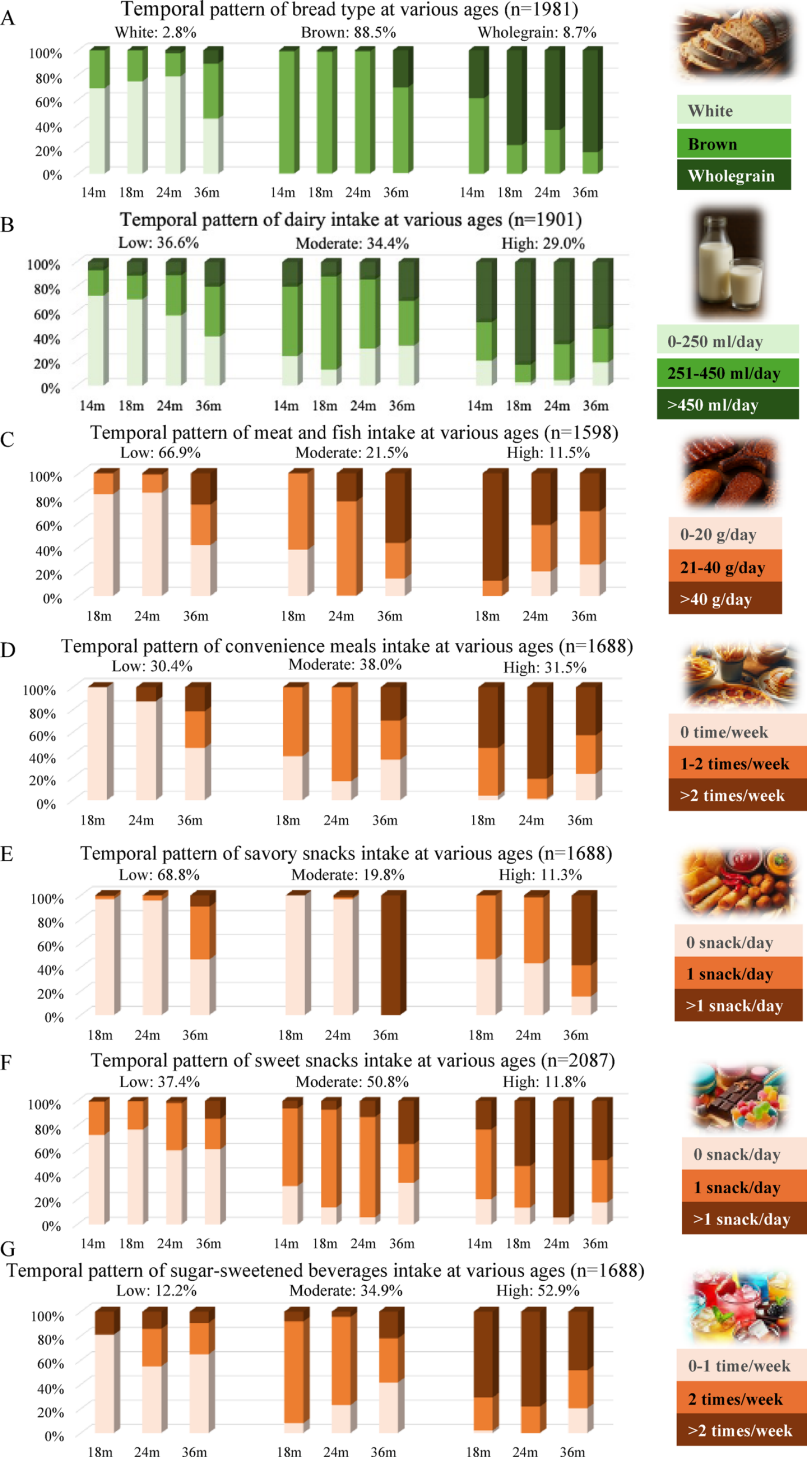
Dietary intake temporal patterns for other food groups showing the percentage of children with low, medium, or high intakes at each age from ages 14 months to 3 years

The results showed that BIC and entropy values were virtually identical for the 3- and 4-class model for the main food intake temporal patterns and the 2- and 3-class model were virtually identical for the jarred/fresh food intake temporal patterns. However, based on additional fit criteria and interpretability, we choose the most optimal model (Supplementary Table S4). For the main food groups, this was the 3-class model, and the 2-class model for the jarred/fresh food groups. The highest posterior probability for each latent class of dietary temporal patterns, following individual assignment, is reported in Supplementary Table S5. The class membership and item response probability parameter estimates for different dietary temporal patterns are reported in Figs. 1 and 2. The dietary intake temporal patterns were labelled after LCA was performed. For fruit, vegetable, dairy, meat and fish, convenience meals, savory snacks, sweet snacks, and SSB, the temporal patterns were categorized as “Low intake,” “Moderate intake,” and “High intake.” The 2-class model for jarred/fresh food groups was labeled as “Low intake” and “High intake.” For bread types, the 3-class model identified “primarily white bread,” “primarily brown bread,” and “primarily wholegrain bread.”

For total fruit, the low fruit intake temporal pattern was dominant (48.2%), with moderate and high fruit intake temporal patterns each representing approximately a quarter of the total of children (Fig. 1A). Across all temporal patterns, a slight downward trend in fruit intake was observed with increasing age. For total vegetable, 54.2% of the children followed a moderate intake temporal pattern, with a decreasing trend across all three temporal patterns when children became older (Fig. 1D). For jarred fruit (Fig. 1B), 45.4% of the children followed the low intake temporal pattern and the rest followed the high intake pattern, and vice versa for fresh fruit (Fig. 1C). In contrast, for jarred vegetable (Fig. 1E), 36.8% of the children followed the low intake temporal pattern and 63.2% followed the high intake pattern. For fresh vegetable (Fig. 1F), the percentage of children in different temporal patterns was reversed. Over time, there was a significant decrease in the intake of jarred fruit and vegetable and an increase in the intake of fresh fruit and vegetable.

For bread type (Fig. 2A), the “primarily brown bread” temporal pattern was the most prevalent (88.5%). With increasing age, the consumption of primarily both brown and wholegrain bread increased in all temporal patterns. Figure 2B indicates that children were relatively evenly distributed across the three identified dairy intake temporal patterns, with a slight increasing trend throughout early childhood. Figure 2C shows that approximately two-thirds of children followed a temporal pattern characterized by low intake of meat and fish during early childhood. Among those with initially low intake, meat and fish intakes tended to increase over time. In Fig. 2D, it shows that children were evenly distributed among the three ‘convenience meals’ temporal patterns, and an increasing trend over age in convenience meals consumption was observed across all temporal patterns. Figures 2E and 2F show that for snacks, the low savory snacks intake temporal pattern was most prevalent (68.8%), and an increasing trend over age in savory snacks intake was observed in all three temporal patterns. Half of the children exhibited a moderate sweet snacks intake temporal pattern (50.8%), characterized by an increasing trend in sweet snacks intake during the measurement period, with some children transitioning from a low or moderate to a high intake level. The dominant temporal pattern of SSB (Fig. 2G) was the high intake pattern (52.9%), while 34.9% of the children followed the moderate intake pattern. Across all temporal pattern groups, SSB intake exhibited a generally stable pattern with a slight decline over time.

### Early childhood dietary temporal patterns and intake at 3 and 10/11 years

Overall, the higher intake dietary temporal patterns were associated with greater consumption of the food group of interest at both 3 and 10 years of age. To be more specific, at 10/11 years of age, 56% of children from the ‘Primarily brown bread’ pattern continued to predominantly consume brown bread, while 70% of those in the ‘Primarily wholegrain bread’ pattern maintained a preference for wholegrain bread (Table 1). Children in the ‘high’ intake pattern for fruit, vegetable and dairy at a young age, exhibited 44%, 37% and 19% higher daily consumption in grams at 10/11 years, respectively, than those in the ‘low’ pattern. For fruit and vegetable, mainly the consumption of fresh produce was associated with a higher intake of fruit or vegetable at the age of 10/11 years. Similarly, for the unhealthy food groups, the ‘high’ intake pattern group consumed 6% more meat and fish, 18% more convenience meals, 9% more savory snacks, 41% more sweet snacks, and 85% more SSB compared to the ‘low’ pattern group at age 10/11 years, with SSB showing the largest disparity (Table 2).

**Table 1:** Temporal patterns of bread type consumed in early childhood in relation to bread type consumed at ages 3y and 10/11y assessed by FFQ

Bread type	“Mainly white”	“Mainly brown”	“Mainly wholegrain”	*P* value	“Mainly white”	“Mainly brown”	“Mainly wholegrain”	*P* value
	At 3 years (n = 1059)				At 10/11 years (n = 745)			
White	10 (40.0%) ^bc^	6 (0.6%) ^a^	0 (0.0%) ^a^	< 0.001	2 (15.4%) ^bc^	19 (2.8%) ^a^	0 (0.0%) ^a^	< 0.001
Brown	12 (48.0%) ^c^	659 (69.4%) ^c^	11 (13.1%) ^ab^		8 (61.5%)	374 (56.0%) ^c^	19 (29.7%) ^b^	
Wholegrain	3 (12.0%) ^c^	285 (30.0%) ^c^	73 (86.9%) ^ab^		3 (23.1%) ^c^	275 (41.2%) ^c^	45 (70.3%) ^ab^	

FFQ: food frequency questionnaire

Data is shown in frequency (percentage). For bread type, chi-square test was conducted. For multiple comparisons, post hoc tests were conducted using Bonferroni adjustment. Superscript letters denote significant differences in column proportions at the 0.05 level: "a" indicates a significant difference from the first column group, "b" indicates a significant difference from the second column group, and "c" indicates a significant difference from the third column group

**Table 2:** Temporal pattern of food intake in early childhood in relation to the same foods at ages 3y and 10/11y assessed by FFQ

Food groups	N	“Low intake”	“Moderate intake”	“High intake”	*P* value	N	“Low intake”	“Moderate intake”	“High intake”	*P* value
	At 3 years					At 10 years				
Intake of fruit and vegetable (g/day)										
Fruit	1243	111(43–167)^bc^	168(111–228)^a^	167(78–228)^a^	< 0.001	852	77(43–223)^bc^	111(77–223)^a^	111(44–223)^a^	< 0.001
Jarred Fruit	1121	116(77–228)	n.a	112(43–224)	< 0.001	769	111(56–223)	n.a	77(43–223)	< 0.001
Fresh Fruit	1121	111(43–224)	n.a	116(77–228)	< 0.001	769	77(43–223)	n.a	111(75–223)	< 0.001
Vegetable	1244	28(6–50)^bc^	51(20–99)^a^	57(21–126)^a^	< 0.001	856	57(21–127)^bc^	65(26–148)^a^	78(39–171)^a^	< 0.001
Jarred Veg	1126	46(19–103)	n.a	41(11–85)	< 0.001	780	68(28–146)	n.a	62(24–142)	0.108
Fresh Veg	1126	40(11–85)	n.a	47(20–103)	< 0.001	780	60(24–140)	n.a	72(29–150)	0.004
Dairy	1169	135(0–407)^bc^	193(11–445)^ac^	303(36–557)^ab^	< 0.001	699	125(0–373)^c^	150(0–465)	149(0–466)^a^	0.013
Meat and fish	1143	45(21–76)^b^	66(41–100)^ac^	48(32–72)^b^	< 0.001	635	69(31–120)^b^	77(44–128)^a^	73(44–114)	0.004
Meat	1143	39(17–69)^b^	57(32–91)^ac^	41(24–67)^b^	< 0.001	635	60(24–108)^b^	70(35–112)^a^	65(36–107)	0.002
Fish	1143	4(0–12)^bc^	7(0–18)^a^	5(0–17)^a^	< 0.001	635	7(0–21)	7(0–21)	7(0–21)	0.517
Convenience meals	1163	25(7–50)^bc^	27(9–56)^ac^	35(16–63)^ab^	< 0.001	673	72(31–122)^c^	80(34–127)	85(45–139)^a^	< 0.001
Savory Snacks	1163	3(1–7)^bc^	12(9–20)^ac^	11(5–22)^ab^	< 0.001	673	11(3–25)^b^	15(6–30)^a^	12(3–37)	< 0.001
Sweet Snacks	1204	23(0–48)^bc^	36(0–73)^ac^	46(26–94)^ab^	< 0.001	772	41(14–83)^bc^	47(25–87)^ac^	58(29–106)^ab^	< 0.001
SSB	1163	47(14–110)^c^	82(28–192)^c^	116(43–318)^ab^	< 0.001	673	101(19–363)^c^	145(33–327)^c^	187(51–428)^ab^	< 0.001

FFQ, food frequency questionnaire; SSB, sugar-sweetened beverages; n.a., not applicable. Data is shown in Median (P10–P90).

For the main food groups, non-parametric Kruskal-Wallis Test was employed. For the jarred/fresh food groups, non-parametric Mann-Whitney U Test was employed. Superscript letters denote significant differences in column proportions at the 0.05 level: "a" indicates a significant difference from the first column group, "b" indicates a significant difference from the second column group, and "c" indicates a significant difference from the third column group.

### Early childhood dietary patterns and diet quality indices at 3 and 10/11 Years

Similarly, the early childhood temporal patterns were associated with overall diet quality indices at 10/11 years (Table 3). After adjusting for confounding factors, virtually all individual food groups showed a dose-dependent association with overall diet quality. The individual associations were weak, and slightly stronger for the DASH score than for the LLDS, and both stronger than for the MDS. The higher intake temporal patterns of healthy foods in early childhood showed higher overall diet quality in later childhood, while higher intake temporal patterns of unhealthy foods showed lower overall diet quality. For both fruit and vegetable, fresh produce was consistently and significantly related to a higher LLDS (for fruit, b = 1.68, 95% CI 0.82 to 2.54; for vegetables, b = 1.46, 95% CI 0.58 to 2.34)) and DASH (for fruit, b = 1.44, 95% CI 0.84 to 2.03; for vegetables, b = 1.28, 95% CI 0.66 to 1.89) scores at 10/11 years of age, whereas jarred fruit and vegetable were related to lower diet quality. The wholegrain bread pattern (LLDS b = 4.69, 95% CI 1.13 to 8.24; DASH b = 4.58, 95% CI 2.12 to 7.05) and the high intake of convenience meals (LLDS b = − 2.78, 95% CI − 3.97 to − 1.60; DASH b = − 1.69, 95% CI − 2.52 to − 0.85) showed the strongest associations for LLDS and DASH. In the moderate intake pattern for savory snacks, children had the highest intake of these foods at ages 3 and 10/11 years, and the lowest LLDS (b = − 2.22, 95% CI − 3.32 to − 1.12) and DASH (b = − 1.78, 95% CI − 2.55 to − 1.02) scores. Children in the high dairy intake pattern group had the lowest scores on the MDS (b = − 0.32, 95% CI − 0.57 to − 0.06). In contrast, no significant associations were observed for dietary intake temporal patterns of meat and fish with overall diet quality at 10/11 years.

**Table 3:** Association between early childhood dietary intake temporal patterns from infancy to 3 years across food groups and diet quality score at 10/11 years

	“Mainly white”/ “Low intake”	“Mainly brown”/ “Moderate intake”		“Mainly wholegrain”/ “High intake”	
Lifelines diet score (LLDS)					
Fruit	Ref	1.42*	(0.39, 2.46)	1.33*	(0.38, 2.29)
Jarred fruit	Ref	n.a	n.a	− 1.53*	(− 2.39, − 0.66)
Fresh fruit	Ref	n.a	n.a	1.68*	(0.82, 2.54)
Vegetable	Ref	0.82	(− 0.06, 1.71)	1.74*	(0.30, 3.18)
Jarred vegetable	Ref	n.a	n.a	− 1.16*	(− 2.01, − 0.30)
Fresh vegetable	Ref	n.a	n.a	1.46*	(0.58, 2.34)
Bread type	Ref	1.85	(− 1.42, 5.13)	4.69*	(1.13, 8.24)
Dairy	Ref	0.51	(− 0.56, 1.58)	− 0.29	(− 1.42, 0.83)
Meat and fish	Ref	− 0.42	(− 1.51, 0.66)	− 1.59	(− 3.36, 0.17)
Convenience meals	Ref	− 0.96	(− 1.99, 0.07)	− 2.78*	(− 3.97, − 1.60)
Savory Snacks	Ref	− 2.22*	(− 3.32, − 1.12)	− 1.48	(− 3.25, 0.30)
Sweet Snacks	Ref	− 0.79	(− 1.73, 0.14)	− 2.20*	(− 3.72, − 0.68)
Sugar− sweetened beverages	Ref	0.42	(− 0.96, 1.80)	− 1.68*	(− 3.04, − 0.33)
Dietary Approaches to Stop Hypertension diet score (DASH)					
Fruit	Ref	1.36*	(0.63, 2.08)	1.01*	(0.34, 1.69)
Jarred fruit	Ref	n.a	n.a	− 1.37*	(− 1.97, − 0.78)
Fresh fruit	Ref	n.a	n.a	1.44*	(0.84, 2.03)
Vegetable	Ref	0.69*	(0.06, 1.31)	1.94*	(0.92, 2.95)
Jarred vegetable	Ref	n.a	n.a	− 1.01*	(− 1.61, − 0.42)
Fresh vegetable	Ref	n.a	n.a	1.28*	(0.66, 1.89)
Bread type	Ref	2.55*	(0.28, 4.82)	4.58*	(2.12, 7.05)
Dairy	Ref	0.57	(− 0.17, 1.31)	− 0.53	(− 1.31, 0.25)
Meat and fish	Ref	− 0.59	(− 1.35, 0.18)	− 0.38	(− 1.62, 0.86)
Convenience meals	Ref	− 0.82*	(− 1.55, − 0.10)	− 1.69*	(− 2.52, − 0.85)
Savory snacks	Ref	− 1.78*	(− 2.55, − 1.02)	− 1.83*	(− 3.07, − 0.60)
Sweet snacks	Ref	− 0.60	(− 1.25, 0.05)	− 1.25*	(− 2.31, − 0.19)
Sugar-sweetened beverages	Ref	− 0.02	(− 0.99, 0.96)	− 1.10*	(− 2.05, − 0.14)
Mediterranean diet score (MDS)					
Fruit	Ref	0.37*	(0.13, 0.60)	0.24*	(0.02, 0.45)
Jarred fruit	Ref	n.a	n.a	− 0.08	(− 0.27, 0.12)
Fresh fruit	Ref	n.a	n.a	0.11	(− 0.08, 0.31)
Vegetable	Ref	0.21*	(0.01, 0.41)	0.32*	(0.00, 0.65)
Jarred vegetable	Ref	n.a	n.a	− 0.15	(− 0.35, 0.04)
Fresh vegetable	Ref	n.a	n.a	0.12	(− 0.08, 0.32)
Bread type	Ref	0.11	(− 0.65, 0.86)	0.49	(− 0.33, 1.30)
Dairy	Ref	− 0.12	(− 0.37, 0.12)	− 0.32*	(− 0.57, − 0.06)
Meat and fish	Ref	0.05	(− 0.20, 0.30)	0.04	(− 0.45, 0.37)
Convenience meals	Ref	− 0.11	(− 0.35, 0.13)	− 0.40*	(− 0.68, − 0.12)
Savory snacks	Ref	− 0.18	(− 0.44, 0.08)	− 0.10	(− 0.51, 0.32)
Sweet snacks	Ref	− 0.23*	(− 0.44, − 0.02)	− 0.54*	(− 0.88, − 0.19)
Sugar-sweetened beverages	Ref	− 0.26	(− 0.58, 0.07)	− 0.27	(− 0.59, 0.05)

*P<0.05. n.a.: not applicable. Data is shown in b (95% CI). Regression coefficients (b) represent the mean difference in diet quality score compared to the reference category. For bread, “Mainly white” temporal pattern was considered the reference category. For other food groups, “Low intake” patterns were considered the reference. Model was adjusted for children’s age and sex, maternal education, mother smoked during pregnancy, and the presence of siblings at birth.

A sensitivity analysis was performed by excluding the food group of the dietary pattern from the diet quality score to assess whether the observed impacts of dietary intake temporal patterns were independent of the food group of interest (Supplementary Table S6). After adjusting for potential confounders, the associations for overall fruit and vegetable patterns were attenuated, whereas the impacts of jarred fruit/vegetable and fresh vegetable intake remained highly consistent with previous findings. No associations were detected for meat and fish patterns in either the primary or sensitivity analyses.

### Association between dietary temporal patterns and family characteristics

**Fig. 3:**
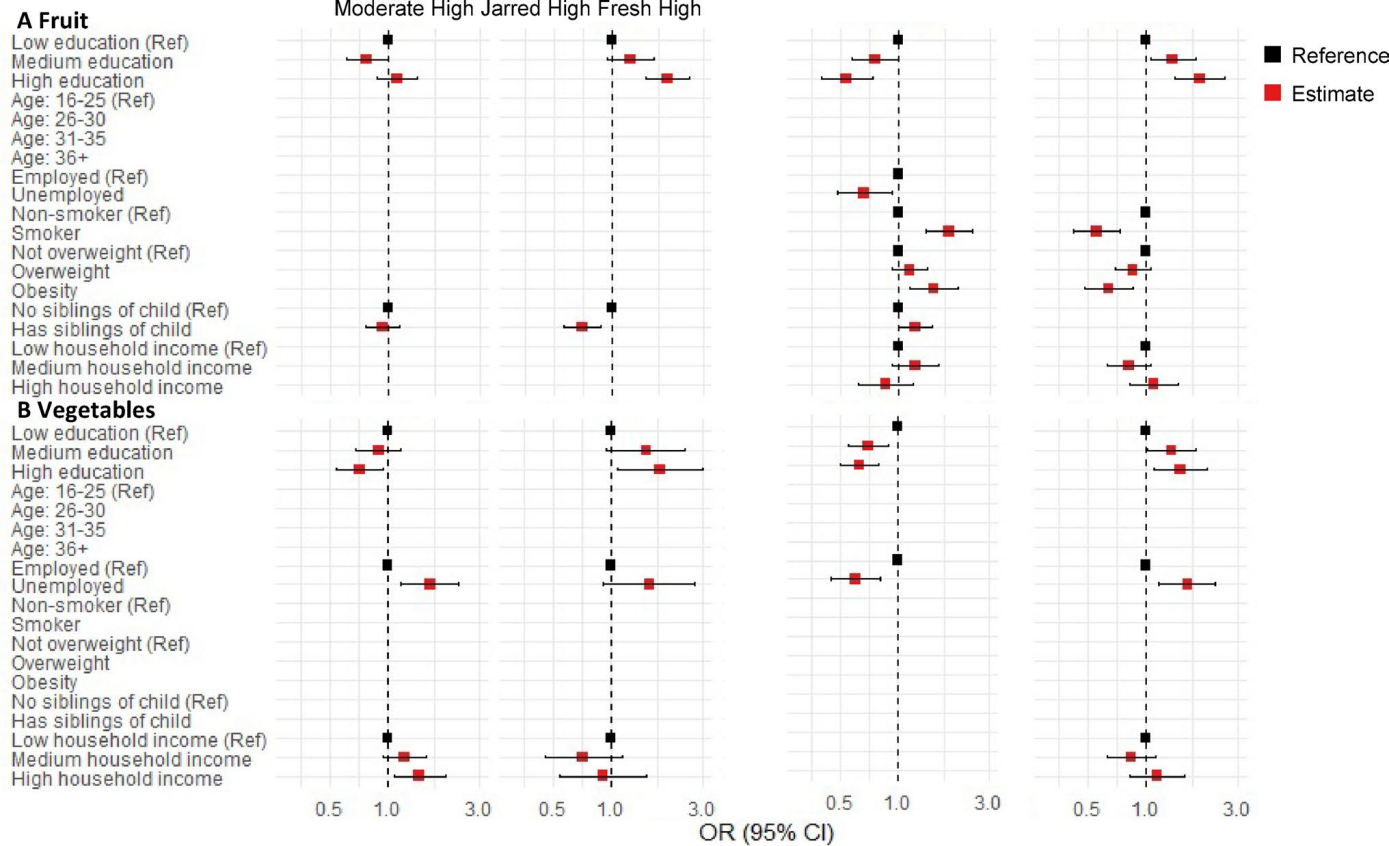
Association between early childhood fruit and vegetable intake temporal patterns and maternal and family characteristics at baseline. “Low intake” temporal patters were considered the reference group. All variables in the regression model were mutually adjusted for one another. To enhance clarity, only variables retained in the final model based on the selection criteria are presented in the figure

**Fig. 4:**
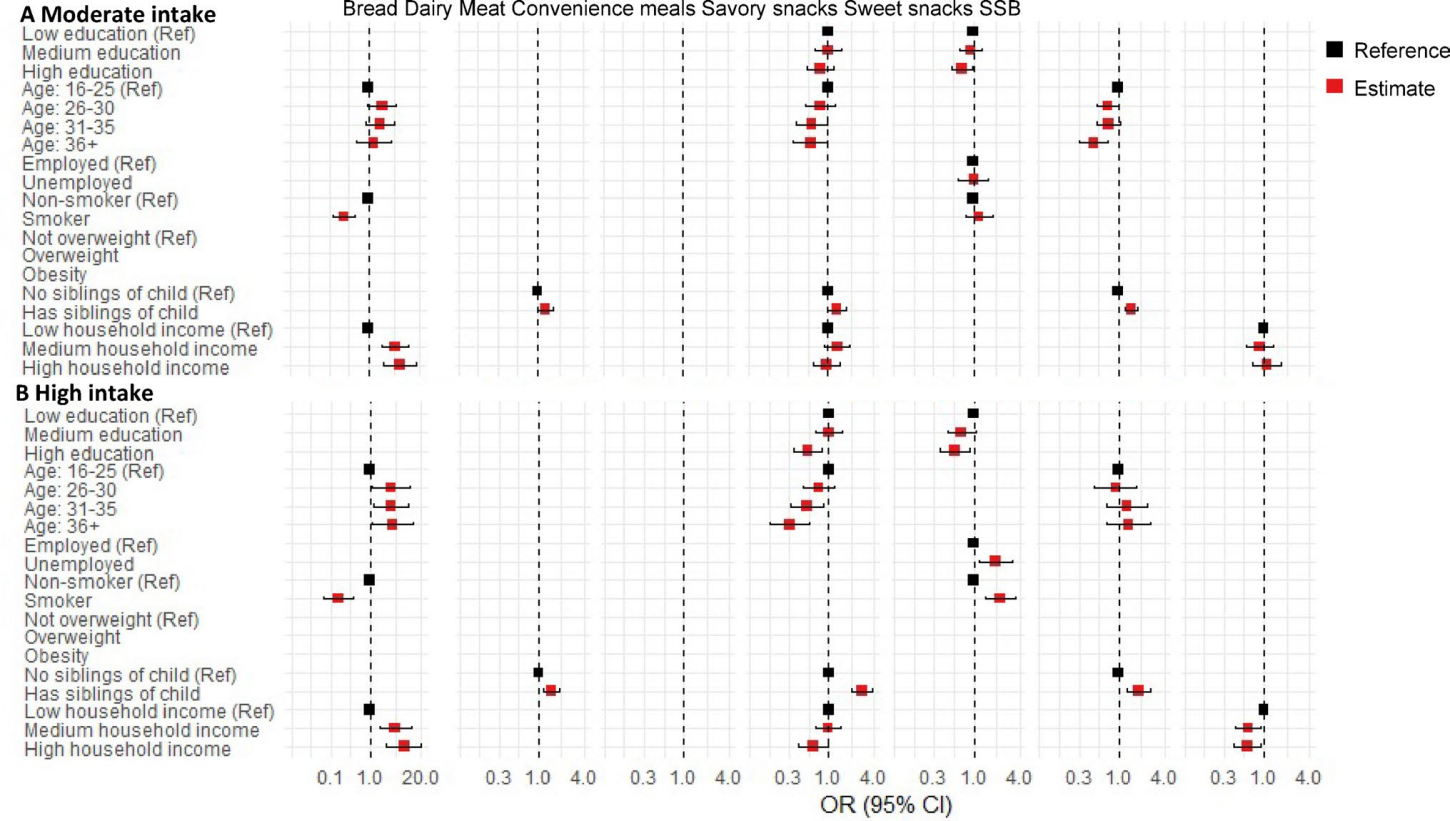
Association between early childhood other food groups intake temporal patterns and maternal and family characteristics at baseline. For bread, “Mainly white” temporal patterns was considered the reference category. For other food groups, “Low intake” temporal patterns were considered the reference category. All variables in the regression model were mutually adjusted for one another. To enhance clarity, only variables retained in the final model based on the selection criteria are presented in the figure.

The relationships between the dietary intake temporal patterns of children and maternal characteristics, and paternal characteristics are presented in Figs. 3 and 4, supplementary Figs. S3 and S4, respectively. Higher levels of parental education and household income, older parental age, and non-smoking status during pregnancy were associated with healthier dietary intake temporal patterns. Additionally, higher parental education level was correlated with increased consumption of fresh produce. Maternal employment was associated with higher consumption of jarred produce, lower consumption of fresh produce, and reduced intake of savory snacks. While paternal employment status was associated with only the type of bread consumed. Higher parental BMI was associated with greater adherence to temporal patterns characterized by high intake of jarred fruit, low intake of fresh fruit, and high intake of convenience meals. Furthermore, being the firstborn or having no siblings was associated with healthier dietary temporal patterns, with the exception of a lower intake pattern for dairy. SSB consumption was associated solely with household income, with higher-income households reporting lower SSB consumption.

## Discussion

Using LCA, we identified dietary intake temporal patterns across nine main food groups and four subgroups from 7 months to 3 years in a Dutch birth cohort from the northern Netherlands. This provides a detailed characterization of early childhood dietary intake temporal patterns specific to this population. The current analysis illustrates the additional value of the use of LCA in capturing dynamic longitudinal changes that cross-sectional studies cannot adequately address. We observed that for all temporal patterns, intake of healthy foods (fruit and vegetable) declined with age, whereas dairy intake showed a modest increase over time. In contrast, the consumption of unhealthy foods (meat and fish, convenience meals, savory snacks, sweet snacks, SSB) increased up to the age of 3 years. Overall, the higher intake temporal patterns of healthy foods were correlated with better diet quality in mid-childhood (10/11 years), whereas higher intake temporal patterns of unhealthy foods showed inverse associations. Furthermore, dietary intake temporal patterns varied significantly with family characteristics. In general, unhealthier food choices were associated with parents who were younger, smoked during pregnancy, had lower education levels, had lower household income, had a higher BMI, and already had other children at the time of birth. These findings show that early food intake track into later childhood, and that these are highly depending on parental characteristics. However, substantial uncertainty in class assignment indicates that these findings are preliminary and should be interpreted with caution. Nevertheless, the findings highlights the critical role of establishing healthy eating habits from the start of complementary feeding in high-risk populations, and stresses the relevance of continuously reinforcing these practices throughout early childhood.

An age-related increase in the consumption of diverse food groups was observed, consistent with the transition from complementary feeding to the family diet, and likely reflecting growing energy demands, food autonomy, and a shift toward solid foods. During infancy, complementary feeding predominantly consisted of fruit and vegetable. With increasing age, children’s diets incorporated more diverse foods, they started to include dairy and SSB, as well as a wide range of solid and high-energy foods, such as bread, meat and fish, convenience meals, and snacks. Bread was identified as one of the most frequently consumed lunch items among Dutch primary school-aged children [46]. Our data shows that, a consumption/selection of brown and wholegrain bread is prominent in the diets of Dutch children, with 89% following a brown bread pattern and 9% preferring wholegrain bread. This contrasts with English children’s growing preference for white bread, as reported in the Avon Longitudinal Study of Parents and Children [47]. In the Netherlands, cultural emphasis, health campaigns, and government initiatives promoting wholegrain consumption have supported healthy bread choices, positioning the country as a model for Europe.

What is of great concern is that we observed major shifts from healthy to less healthy diets already during early childhood. This is consistent with findings from the Generation R study, which reported that by age 2 years, more children consumed greater energy from unhealthy taste clusters compared to age 1 year [48]. In a separate Avon Longitudinal Study of Parents and Children examining children between 18 and 43 months of age, their finding showed a progressive shift away from nutrient-rich foods such as vegetables and beef, accompanied by increased consumption of energy-dense, nutrient-poor items [15]. Similarly, the Healthy Smiles Healthy Kids birth cohort, which tracked children from 4 months to 3 years of age [49], showed a decline in core foods intake (including fruit and vegetable) and an increase in discretionary foods (including potato chips, savory snacks, sweet snacks, and SSB). Increased consumption of energy-dense and nutrient-poor foods contributes ‘empty calories’ to young children’s diets [12], replacing foods of higher nutritional quality and value [50]. This trend emphasizes the importance of sustained family nutrition education as children gain dietary independence. Targeted interventions are crucial for supporting families most at risk of unhealthy dietary patterns, ensuring healthier eating habits are maintained during early childhood.

All dietary intake temporal patterns in this study were strongly associated with later food consumption, consistent with prior research suggesting early-life dietary patterns tend to persist into later life [2, 3, 11, 13, 14]. However, as yet only limited data was available to quantify this association. We found that Children in the ‘high’ healthy food intake pattern consumed 37–44% more fruits and vegetables, and 19% more dairy at age 10–11 years compared to those in the ‘low’ intake pattern, while those in the ‘high’ unhealthy food pattern consumed 6–85% more meat and fish, convenience meals, snacks, and sugar-sweetened beverages, with the largest disparity in SSB intake. Most children within the primarily bread-type temporal patterns maintained these eating patterns over time. Notably, these dietary habits persisted into later childhood, indicating the stability of dietary habits over time. In addition to food intake, dietary temporal patterns were also associated with overall diet quality in later childhood, further emphasizing their implications for longer-term diet. More specifically, regular consumption of brown and wholegrain bread, fruit, and vegetables at early age correlated with higher diet quality at age 10/11 years. In contrast, frequent consumption of dairy, convenience meals, savory snacks, sweet snacks, and SSB correlated with poorer diet quality. The lack of association between dairy intake temporal patterns and later LLDS and DASH scores may reflect the generally high dairy consumption in the Netherlands, where most individuals already adhere to national dietary guidelines for dairy. In contrast, high dairy intake is considered an unfavorable component in the MDS, which may explain its negative association. Without detailed information on dairy types, e.g. fat level, or added sugars, it is difficult to determine whether higher intake reflects also healthier choices. No associations were found for meat and fish intake temporal patterns. Existing literature presents mixed findings: a U.S. cross-sectional study in 10-year-olds reported borderline positive associations between overweight status and total meat intake (including mixed meats, poultry, seafood, eggs, pork, and beef) [51], while a Korean cross-sectional study found a higher risk of being overweight or obesity among children who did not consume meat [52]. These inconsistencies highlight the need for further research, particularly on the effects of different types of meat.

Consumption of fresh fruit and vegetable is associated with higher intake of these foods and better overall diet quality in later childhood, while the opposite was true for jarred produce. This is consistent with the findings of the Avon Longitudinal Study of Parents and Children, which reported that feeding fresh fruits and vegetables at 6 months of age was associated with higher consumption of these foods at 7 years compared with feeding jarred or packet baby food versions [53], and frequent consumption of commercial baby foods from jars, packets, and cans between 6 and 24 months of age correlated with less healthy transitional diets [54]. While jarred produce offered convenience and soft texture, studies indicated that it may not enhance nutrient density in children’s diets [55] or encourage preferences for novel flavors, which is important to increase the intake of vegetable [56]. In contrast, fresh foods provide diverse flavors and textures, promoting broader food acceptance in children. However, a previous cross-sectional study reported that canned fruit and vegetable intake was related to a better overall diet quality compared to non-consumers [57]. Although canned and jarred foods can offer nutritional benefits, including a variety of carotenoids, vitamins, minerals, and fiber [58, 59], our findings show that fresh fruit and vegetables may be more beneficial for fostering healthier eating habits in later childhood. Complementary feeding is a critical period for shaping taste preferences, with varied flavor exposure encouraging acceptance of nutrient-rich foods [60, 61]. Consistent introduction of healthy foods during this period can foster healthier eating habits later in life [53], whereas repeated exposure to unhealthy foods may have adverse effects [62]. These findings highlight the need for early interventions from infancy to establish healthy dietary practices.

Family characteristics, particularly maternal factors, play a critical role in shaping children’s diets. As hypothesized, higher unhealthy food intake was associated with lower maternal education and household income. However, maternal employment showed inconsistent findings, related to an unhealthy diet (increased intake of jarred fruit and vegetable and reduced consumption of fresh fruit and vegetable), and to healthy eating (lower consumption of savory snacks). Previous studies linked low maternal education to diets high in energy, sugar, and fat, and low in fiber [13, 49], while higher household income is associated with healthier diets, including increased fruit and vegetable intake [43, 44]. The impact of maternal employment varies geographically: in China, it correlates with healthier eating behaviors [63], and in Japan, extended working hours reduced confectioneries consumption [64]. Conversely, UK studies associated maternal employment with increased SSB intake and reduced fruit and vegetable consumption among children [65]. Our study also highlights the potential influence of paternal characteristics on children’s diets, an area often overlooked [66]. Parental education may be related to family nutrition knowledge, while income influences purchasing behaviors, thus shaping children’s dietary intake temporal patterns. Socioeconomic impact underscores the need for policies like subsidized nutrition programs to promote healthy eating across diverse cultural and socio-economic backgrounds.

Parental health behaviors further influence dietary intake temporal patterns. Children of parents who had a high BMI were more likely to consume convenience meals and jarred fruit and less likely to eat fresh fruit. This aligns with studies from Australia, France, and the U.S., where higher maternal BMI was linked to less healthy feeding practices and greater child autonomy over food choices, often leading to unhealthy dietary patterns [67–69]. These findings are concerning given that in 2022, global data indicated 43% of adults were overweight, with 16% living with obesity [70]. Parental smoking during pregnancy predicted unhealthy dietary intake temporal patterns. Data from New Zealand cohorts [71–73] showed that maternal smoking correlated to higher intake of energy-dense, sugary, and high-fat foods. These results highlight the need for targeted interventions addressing parental health and behavior.

Beyond SES and health-related lifestyle factors, other family characteristics also shape children’s diets. Younger parental age was associated with lower wholegrain bread intake and higher convenience meals and sweet snacks consumption, aligning with previous studies [49, 54, 71] showing older maternal age correlated with healthier child diets, due to greater health emphasis and nutritional knowledge. More interesting is that the presence of siblings at birth was related to unhealthier dietary temporal patterns. Evidence from the Healthy Smiles Healthy Kids study [49] and the EDEN mother–child cohort [13] revealed that multiparity resulted in reduced consumption of core foods and greater reliance on discretionary items. The challenges of meal preparation in larger families and the sharing of discretionary foods among siblings likely contribute to these patterns [74].

### Strengths and limitations

The current study is unique in the fact that data was used from infancy to middle childhood of the same child. Multiple food intakes were repeatedly measured throughout early childhood, providing a robust and reliable representation of dietary temporal patterns within very young Dutch children. This study explores the dietary intake temporal patterns for nine specific food groups, including some typical eating habits of the Dutch during the first three years of life, for example, the choice of bread type, the use of ready-to-eat adjusted baby foods, and convenience meals such as pizza, fries (patat), and pasta. Moreover, LCA was applied to identify dietary intake temporal patterns, capturing dynamic changes in children’s food intakes. To our knowledge, no studies have applied LCA to dietary intake temporal patterns in early childhood. LCA enables detailed examination of individual information, including changes in intake levels over time and shifts in dietary behavior. Additionally, the large sample size retained for the pattern analysis (n = 2552) resulted in a high precision and power of our findings.

However, several limitations should also be acknowledged. First, the use of different dietary assessment questionnaires across time points posed challenges for harmonizing intake data and constructing consistent patterns. To enhance comparability, food intakes were categorized during pattern modeling. Notably, the FFQ at age 3 years captured more detailed intake information than at other ages, potentially reducing consistency across time points. However, this greater detail may have contributed to more realistic patterns. Improved dietary detail at earlier ages may have strengthened observed associations, particularly for savory snacks and meat and fish intake temporal patterns. Second, children’s dietary intake was reported by their parents, which may introduce potential biases, including under-reporting, social desirability bias, and inaccurate dietary recall. Third, while we adjusted for several confounders, residual confounding by unmeasured factors (e.g., biological and environmental influences) cannot be entirely ruled out. Fourth, latent class membership was determined using highest posterior probability, and classification uncertainty was not incorporated into subsequent analyses. As a result, associations involving class membership may be attenuated or biased, and the findings should be interpreted as preliminary. Finally, although the sample size for the pattern analysis was large, loss to follow-up in diet quality assessments at age 10/11 years may have attenuated the observed associations.

## Conclusion

This study used LCA to examine dynamic changes in children's dietary intake temporal patterns from 7 months to 3 years, revealing key patterns in their eating behaviors. Healthy food consumption (fruit and vegetable) declined with age, while intake of unhealthy foods (meat and fish, convenience meals, savory and sweet snacks, and SSB) increased. Dietary variety expanded with the transition to solid foods, presumably reflecting growing energy demands and autonomy over food choices. Dutch children showed high consumption of healthier bread types, with the majority following brown and wholegrain bread temporal patterns, this may reflect cultural preferences and policy influences.

The high intake temporal patterns of healthy foods in early childhood, were associated with better diet quality, whereas high intake temporal patterns of unhealthy foods were associated with poorer diet quality at ages 10–11 years. These findings emphasize the importance of complementary and early childhood feeding practices that promote varied and nutrient-rich foods.

SES and family characteristics influenced children’s dietary intake temporal patterns. As expected, SES factors, such as higher parental education and household income, were associated with healthier diets, highlighting the need for nutritional assistance and education across diverse socioeconomic backgrounds. Furthermore, we identify younger parental age, higher parental BMI, and smoking during pregnancy as risk factors correlated with less healthy dietary patterns, indicating the potential value of targeted nutrition counseling for high-risk families. Interestingly, the influence of siblings on children’s diets underscores the continued need for tailored family-based dietary interventions, particularly in households with two or more children.

## Supplementary Information

Below is the link to the electronic supplementary material.

Supplementary Material 1

## Data Availability

This longitudinal study used the data from the GECKO Drenthe birth cohort. Data described in the manuscript, code book, and analytic code will be made available upon reasonable request.

## Abbreviations

AIC: Akaike information criterion
ANOVA: Analysis of variance
BIC: Bayesian information criterion
BMI: Body Mass Index
DASH: Dietary approaches to stop hypertension
EHII: Equivalized household income indicator
FFQ: Food frequency questionnaire
GECKO: Groningen expert center for kids with obesity
LCA: Latent class analysis
LLDS: Lifelines diet score
MDS: Mediterranean diet score
SES: Socioeconomic status
SSB: Sugar-sweetened beverages
