# Supplementary material for: Dietary intake temporal patterns during early childhood in relation to diet quality in middle childhood and the role of family characteristics: the GECKO Drenthe cohort
Source: Eur J Nutr. 2026 Mar 9;65(3):89. doi: 10.1007/s00394-026-03934-8 (PMC12971947; doi:10.1007/s00394-026-03934-8)
Supplement: Supplementary file 1 — Supplementary Material 1 [file 394_2026_3934_MOESM1_ESM.docx]

Fig. S1 Flowchart of participant enrollment

Aim1

born

7months to 3years

10/11 years

Children with 10/11y FFQ data

n=856

Relate to daily intake and diet quality at 10/11y

Fruit: n=852

Jars/fresh fruit: n=769

Vegetable: n=856

Jars/fresh vegetable: n=780

Bread type: n=745

Dairy: n=699

Meat and fish: n=635

Convenience meals: n=673

Savory snacks: n=673

Sweet snacks: n=772

SSB: n=673

Children participated at birth

n=2842

Children with valid data during follow-up in early childhood

n=2684

Missing during follow-up in early childhood

n=158 excluded

Children with only 1 measurement of food intake

n=132 excluded

Children with at least 2 measurements of food intake

n=2552

Children with

dietary intake temporal patterns

Fruit: n=2517

Jars/fresh fruit: n=2229

Vegetable: n=2551

Jars/fresh vegetable: n=2284

Bread type: n=1981

Dairy: n=1901

Meat and fish: n=1598

Convenience meals: n=1688

Savory snacks: n=1688

Sweet snacks: n=2087

SSB: n=1688

Missing during follow-up at 10/11 years

n=1696 excluded

Relate dietary intake temporal patterns with family characteristics

Fruit: n=2223

Jars/fresh fruit: n=1985

Vegetable: n=2256

Jars/fresh vegetable: n=2026

Bread type: n=1792

Dairy: n=1622

Meat and fish: n=1375

Convenience meals: n=1541

Savory snacks: n=1541

Sweet snacks: n=1889

SSB: n=1541

Aim3

Aim2

SSB: sugar-sweetened beverages.

Fig. S2 The questionnaires used for daily food intake during early childhood


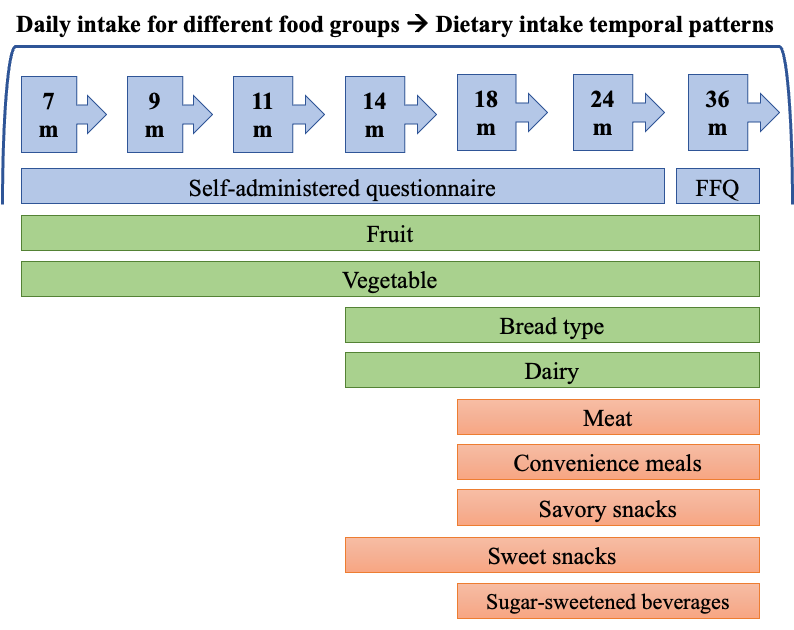


FFQ: food frequency questionnaire.

Table S1 Parental-completed questionnaires investigating food intake from 7 month to 24 month

|  | Type | Questions |
| --- | --- | --- |
| **Fruit at 7, 9, 11, 14m** | Jarred fruit  Fresh fruit | **Amounts:**  jars for jarred per day  pieces for fresh per day |
| **Fruit at 18, 24m** | -no specification - | **Frequency:**  Less than once a day  Once a day  Two times a day  Three times a day  More than three times a day |
| **Vegetable at 7, 9, 11, 14m** | Jarred vegetable  Fresh vegetable | **Amounts:**  jars for jarred per day  tablespoons for fresh per day |
| **Vegetable at 18, 24m** | -no specification - | **Amounts:** tablespoons per day |
| **Bread at 14, 18, 24m** | White  Brown  Wholegrain | Indicate the main type of bread |
| **Dairy at 14, 18, 24m** | -no specification - | **Amounts:**  cups for milk and yogurt drink per day  plates for yogurt per day |
| **Meat and fish at 18, 24m** | -no specification - | **Amounts:**  tablespoons per day |
| **Convenience meals at 18, 24m** | Pizza  Pancakes  Fries | **Frequency:**  Once a month or less  Once every two weeks  Once a week  More than once a week |
| **Savory snacks (e.g. handful of chips, piece of cheese**  **or sausage) at 18, 24m** | -no specification - | **Frequency:**  Less than once a day  Once a day  Two times a day  Three times a day  More than three times a day |
| **Sweet snacks (e.g. cookie or cake, handful candy or smarties, chocolate) at 14, 18, 24m** | -no specification - | **Frequency:**  Less than once a day  Once a day  Two times a day  Three times a day  More than three times a day |
| **Sugar-sweetened beverages at 18, 24m** | Fruit juice  Lemonade | **Frequency:**  Less than once a day  Once a day  Two times a day  Three times a day  More than three times a day |

One jar of jarred fruit = 194 g; one piece of fresh fruit = 120 g; one jar of jarred vegetables = 100 g; one tablespoon of fresh vegetables = 30 g; one tablespoon of ‘meat or fish’ = 20 g. For dairy products, volumes consumed from cups and plates were reported.

Table S2. The ranges and frequency of daily consumption used to categorize low, moderate and high intake.

|  | Low intake | Moderate intake | High intake |
| --- | --- | --- | --- |
| Fruit at 7, 9, 11, 14m | 0-100 g/day | 101-200 g/day | >200 g/day |
| Fruit at 18, 24m | 0 time/day | 1 time/day | >1 time/day |
| Vegetable, all ages | 0-50 g/day | 51-100 g/day | >100 g/day |
| Dairy at 14, 18, 24m | 0-250 ml/day | 251-450 ml/day | >450 ml/day |
| Meat and fish at 18, 24m | 0-20 g/day | 21-40 g/day | >40 g/day |
| Convenience meals at 18, 24m | 0 time/week | 1-2 times/week | >2 times/week |
| Savory snacks at 18, 24m | 0 snacks/day | 1 snacks/day | >1 snacks/day |
| Sweet snacks at 14, 18, 24m | 0 snacks/day | 1 snacks/day | >1 snacks/day |
| Sugar-sweetened beverages at 18, 24 m. | 0-1 time/day | 2 time/day | >2 times/day |

The ranges and frequency for all food groups at 3 years of age (measured with FFQ) were based on tertiles of 3-year food intake.

Table S3 General characteristics of included subjects at the start of the GECKO cohort

| **Characteristics** | Included in dietary intake temporal patterns (n=2552) | Included in 10/11 year follow-up (n=856) |
| --- | --- | --- |
| **Gender** | |  |
| Boys | 1,273 (49.9%) | 413 (48.2%) |
| Girls | 1,279 (50.1%) | 443 (51.8%) |
| **Maternal education during pregnancy** | |  |
| Low | 801 (31.4%) | 105 (12.3%) |
| Medim | 743 (29.1%) | 408 (47.7%) |
| High | 882 (34.6%) | 342 (40.0%) |
| **Paternal education during pregnancy** | |  |
| Low | 989 (38.8%) | 208 (24.3%) |
| Medium | 632 (24.8%) | 329 (38.4%) |
| High | 729 (28.6%) | 301 (35.2%) |
| **Maternal employment status** | |  |
| Employed | 2,192 (85.9%) | 792 (92.5%) |
| Unemployed | 236 (9.2%) | 59 (6.9%) |
| **Paternal employment status** | |  |
| Employed | 2,308 (90.4%) | 827 (96.6%) |
| Unemployed | 53 (2.1%) | 12 (1.4%) |
| **Mother smoked during pregnancy** | |  |
| No | 2,067 (1.0%) | 756 (89.4%) |
| Yes | 338 (13.2%) | 81 (9.5%) |
| **Father smoking during pregnancy** | |  |
| No | 1,380 (54.1%) | 518 (60.5%) |
| Yes | 742 (29.1%) | 226 (26.4%) |
| **Siblings at birth** | |  |
| No | 999 (39.1%) | 346 (40.4%) |
| Yes | 1,473 (57.7%) | 502 (58.6%) |
| **Equivalized Household Income Indicator** | 1,946 (1,466 - 2,561) | 2218 (1595 - 2603) |
| **Mother BMI (kg/m^2^)** | 23.8 (20.0 - 31.0) | 23.6 (19.9 – 29.8) |
| **Father BMI (kg/m^2^)** | 25.6 ± 3.3 | 25.25 ± 3.22 |
| **Mother age (years)** | 31.3 ± 4.4 | 31.13 ± 4.02 |
| **Father age (years)** | 34.1 ± 4.9 | 33.83 ± 4.59 |
| Data is shown in means ± SDs for continuous variables with a normal distribution, medians (P10 - P90) for continuous variables with a skewed distribution, and frequencies and percentages for categorical variables. | | |

Table S4 Fit information for LCA modeling dietary intake temporal patterns during early childhood with 2-5 latent classes

|  | K | AIC | BIC | Entropy | df | Log-Likelihood |
| --- | --- | --- | --- | --- | --- | --- |
| **Fruit** | 2 | 17,408.8 | 17,577.9 | 0.551 | 2157 | -8,675.4 |
|  | 3 | 17,175.5 | 17,432.1 | 0.579 | 2142 | -8,543.7 |
|  | 4 | 17,058.0 | **17,402.1** | **0.592** | 2127 | -8,470.0 |
|  | 5 | 16,996.1 | 17,427.6 | 0.566 | 2112 | -8,424.1 |
| **Jarred Fruit** | 2 | 8,103.1 | **8,200.2** | **0.792** | 63 | -4,034.6 |
|  | 3 | 8,085.8 | 8,234.3 | 0.670 | 54 | -4,016.9 |
|  | 4 | 8,077.4 | 8,277.3 | 0.665 | 45 | -4,003.7 |
|  | 5 | 8,088.2 | 8,339.4 | 0.642 | 36 | -4,000.1 |
| **Fresh Fruit** | 2 | 11,834.9 | 11,932.0 | **0.789** | 63 | -5,900.4 |
|  | 3 | 11,572.7 | **11,721.1** | 0.739 | 54 | -5,760.3 |
|  | 4 | 11,574.3 | 11,774.2 | 0.686 | 45 | -5,752.2 |
|  | 5 | 11,559.2 | 11,810.4 | 0.675 | 36 | -5,735.6 |
| **Vegetable** | 2 | 20,457.3 | 20,626.8 | **0.473** | 2157 | -10,199.7 |
|  | 3 | 20,282.5 | **20,539.6** | 0.411 | 2142 | -10,097.2 |
|  | 4 | 20,222.1 | 20,567.0 | 0.409 | 2127 | -10,052.1 |
|  | 5 | 20,190.6 | 20,623.1 | 0.453 | 2112 | -10,021.3 |
| **Jarred vegetable** | 2 | 8,121.9 | **8,219.3** | **0.681** | 63 | -4,043.9 |
|  | 3 | 8,093.4 | 8,242.5 | 0.617 | 54 | -4,020.7 |
|  | 4 | 8,087.8 | 8,288.5 | 0.651 | 45 | -4,008.9 |
|  | 5 | 8,103.9 | 8,356.1 | 0.511 | 36 | -4,007.9 |
| **Fresh vegetable** | 2 | 11,055.4 | 11,152.9 | **0.677** | 63 | -5,510.7 |
|  | 3 | 10,935.0 | **11,084.1** | 0.636 | 54 | -5,441.5 |
|  | 4 | 10,937.3 | 11,138.0 | 0.588 | 45 | -5,433.6 |
|  | 5 | 10,934.4 | 11,186.7 | 0.551 | 36 | -5,423.2 |
| **Bread type** | 2 | 4,173.7 | 4,268.7 | 0.767 | 63 | -2,069.8 |
|  | 3 | 3,958.6 | **4,104.0** | **0.770** | 54 | -1,953.3 |
|  | 4 | 3,962.3 | 4,158.0 | 0.449 | 45 | -1,946.1 |
|  | 5 | 3,979.8 | 4,225.8 | 0.359 | 36 | -1,945.9 |
| **Dairy** | 2 | 11912.8 | 12007.2 | 0.516 | 63 | -5939.4 |
|  | 3 | 11,855.4 | **11,999.7** | 0.445 | 54 | -5,901.7 |
|  | 4 | 11835.8 | 12030.0 | 0.513 | 45 | -5882.9 |
|  | 5 | 11844.9 | 12089.1 | **0.560** | 36 | -5878.5 |
| **Meat and fish** | 2 | 7237.6 | 7307.5 | 0.627 | 13 | -3605.8 |
|  | 3 | 7,219.2 | **7,219.8** | **0.661** | 6 | -3,589.6 |
|  | 4 | 7232.1 | 7377.2 | 0.445 | -1 | -3589.0 |
|  | 5 | 7244.3 | 7427.1 | 0.438 | -8 | -3588.1 |
| **Convenience meals** | 2 | 85,38.3 | 8,608.9 | 0.639 | 13 | -4,256.1 |
|  | 3 | 8,403.4 | **8,512.0** | **0.852** | 6 | -4,181.7 |
|  | 4 | 8,412.0 | 8,558.7 | 0.813 | -1 | -4,179.0 |
|  | 5 | 8,425.4 | 8,610.1 | 0.654 | -8 | -4,178.7 |
| **Savory snacks** | 2 | 4,398.6 | **4,469.2** | 0.499 | 13 | -2,186.3 |
|  | 3 | 4,406.7 | 4,515.3 | **0.594** | 6 | -2,183.3 |
|  | 4 | 4,419.6 | 4,566.2 | 0.374 | -1 | -2,182.8 |
|  | 5 | 4,433.2 | 4,617.8 | 0.250 | -8 | -2,182.6 |
| **Sweet snacks** | 2 | 11,840.8 | 11,936.7 | 0.508 | 63 | -5,903.4 |
|  | 3 | 11,731.8 | **11,878.5** | **0.544** | 54 | -5,839.9 |
|  | 4 | 11,740.1 | 11,937.6 | 0.529 | 45 | -5,835.0 |
|  | 5 | 11,753.5 | 12,001.8 | 0.477 | 36 | -5,832.7 |
| **Sugar-sweetened beverages** | 2 | 8,250.7 | 8,321.3 | 0.575 | 13 | -4,112.4 |
|  | 3 | 8,207.9 | **8,316.5** | **0.615** | 6 | -4,083.9 |
|  | 4 | 8,214.6 | 8,361.3 | 0.548 | -1 | -4,080.3 |
|  | 5 | 8,225.3 | 8,409.9 | 0.508 | -8 | -4,078.6 |
| LCA: latent class analysis; AIC: Akaike information criterion; BIC: Bayesian information criterion; df: degrees of freedom. | | | | | | |

Table S5 Posterior probility of LCA modeling dietary intake temporal patterns

|  |  |  | Mainly white/ Low intake |  | Mainly brown/ Moderate intake |  | Mainly wholegrain/ High intake |
| --- | --- | --- | --- | --- | --- | --- | --- |
| Fruit | Low intake |  | 0.87 (0.59, 0.99) |  | 0.07 (0.00, 0.33) |  | 0.02 (0.00, 0.31) |
|  | Moderate intake |  | 0.10 (0.01, 0.37) |  | 0.80 (0.56, 0.95) |  | 0.01 (0.00, 0.11) |
|  | High intake |  | 0.01 (0.00, 0.10) |  | 0.06 (0.01, 0.26) |  | 0.95 (0.61, 1.00) |
| Jarred Fruit | Low intake |  | 0.99 (0.83, 1.00) |  | n.a. |  | 0.02 (0.00, 0.21) |
|  | High intake |  | 0.01 (0.00, 0.17) |  | n.a. |  | 0.98 (0.79, 1.00) |
| Fresh Fruit | Low intake |  | 0.98 (0.80, 1.00) |  | n.a. |  | 0.00 (0.00, 0.19) |
|  | High intake |  | 0.02 (0.00, 0.20) |  | n.a. |  | 1.00 (0.81, 1.00) |
| Vegetable | Low intake |  | 0.72 (0.51, 0.91) |  | 0.13 (0.01, 0.40) |  | 0.03 (0.00, 0.25) |
|  | Moderate intake |  | 0.24 (0.07, 0.46) |  | 0.79 (0.54, 0.96) |  | 0.10 (0.01, 0.36) |
|  | High intake |  | 0.01 (0.00, 0.09) |  | 0.02 (0.00, 0.17) |  | 0.82 (0.47, 0.99) |
| Jarred Vegetable | Low intake |  | 0.95 (0.74, 1.00) |  | n.a. |  | 0.02 (0.00, 0.23) |
|  | High intake |  | 0.05 (0.00, 0.26) |  | n.a. |  | 0.98 (0.77, 1.00) |
| Fresh Vegetable | Low intake |  | 0.98 (0.82, 1.00) |  | n.a. |  | 0.04 (0.00, 0.38) |
|  | High intake |  | 0.02 (0.00, 0.18) |  | n.a. |  | 0.96 (0.62, 1.00) |
| Bread type | Mainly white |  | 1.00 (0.68, 1.00) |  | 0.00 (0.00, 0.00) |  | 0.00 (0.00, 0.00) |
|  | Mainly brown |  | 0.00 (0.00, 0.32) |  | 1.00 (0.98, 1.00) |  | 0.07 (0.00, 0.46) |
|  | Mainly wholegrain |  | 0.00 (0.00, 0.00) |  | 0.01 (0.00, 0.02) |  | 0.93 (0.54, 1.00) |
| Dairy | Low intake |  | 0.81 (0.55, 0.97) |  | 0.16 (0.05, 0.41) |  | 0.04 (0.01, 0.23) |
|  | Moderate intake |  | 0.14 (0.03, 0.36) |  | 0.69 (0.50, 0.90) |  | 0.11 (0.02, 0.34) |
|  | High intake |  | 0.01 (0.00, 0.11) |  | 0.07 (0.02, 0.28) |  | 0.83 (0.49, 0.98) |
| Meat and fish | Low intake |  | 1.00 (0.60, 1.00) |  | 0.15 (0.04, 0.37) |  | 0.00 (0.00, 0.07) |
|  | Moderate intake |  | 0.00 (0.00, 0.31) |  | 0.75 (0.62, 0.91) |  | 0.00 (0.00, 0.31) |
|  | High intake |  | 0.00 (0.00, 0.05) |  | 0.04 (0.00, 0.19) |  | 1.00 (0.55, 1.00) |
| Convenience meals | Low intake |  | 1.00 (0.72, 1.00) |  | 0.00 (0.00, 0.00) |  | 0.02 (0.00, 0.09) |
|  | Moderate intake |  | 0.00 (0.00, 0.01) |  | 0.99 (0.90, 1.00) |  | 0.00 (0.00, 0.01) |
|  | High intake |  | 0.00 (0.00, 0.16) |  | 0.01 (0.00, 0.10) |  | 0.98 (0.89, 1.00) |
| Savory Snacks | Low intake |  | 0.96 (0.75, 0.99) |  | 0.23 (0.22, 0.23) |  | 0.12 (0.02, 0.47) |
|  | Moderate intake |  | 0.00 (0.00, 0.22) |  | 0.72 (0.68, 0.72) |  | 0.00 (0.00, 0.07) |
|  | High intake |  | 0.03 (0.01, 0.04) |  | 0.05 (0.05, 0.11) |  | 0.76 (0.48, 0.98) |
| Sweet Snacks | Low intake |  | 0.89 (0.60, 0.99) |  | 0.09 (0.01, 0.40) |  | 0.00 (0.00, 0.10) |
|  | Moderate intake |  | 0.10 (0.01, 0.35) |  | 0.85 (0.55, 0.97) |  | 0.27 (0.06, 0.39) |
|  | High intake |  | 0.00 (0.00, 0.03) |  | 0.00 (0.00, 0.27) |  | 0.73 (0.51, 0.94) |
| Sugar-sweetened beverages | Low intake |  | 0.69 (0.56, 0.93) |  | 0.00 (0.00, 0.07) |  | 0.01 (0.00, 0.07) |
|  | Moderate intake |  | 0.26 (0.06, 0.43) |  | 0.87 (0.67, 1.00) |  | 0.02 (0.00, 0.19) |
|  | High intake |  | 0.02 (0.00, 0.16) |  | 0.13 (0.00, 0.26) |  | 0.96 (0.74, 1.00) |

LCA: latent class analysis. n.a.: not applicable.

Data is shown in Median (P10, P90). Column headers indicate the most likely assigned class; row labels denote posterior probabilities for each possible class.

Table S6 Association between early childhood dietary intake temporal patterns from infancy to 3 years across food groups and diet quality score at 10/11 years (components of interest were excluded from total diet quality score)

|  | “Mainly white”/ “Low intake” |  | “Mainly brown”/  “Moderate intake” | |  | “Mainly wholegrain”/  “High intake” | |
| --- | --- | --- | --- | --- | --- | --- | --- |
|  | |  | b | 95%CI |  | b | 95%CI |
| **LLDS without fruit component** | |  |  |  |  |  |  |
| Fruit | ref |  | 0.67 | (-0.30, 1.65) |  | 0.77 | (-0.13, 1.67) |
| Jarred Fruit | ref |  | n.a. | n.a. |  | -1.16* | (-1.97, -0.35) |
| Fresh Fruit | ref |  | n.a. | n.a. |  | 1.28* | (0.47, 2.09) |
| **LLDS without vegetable component** | |  |  |  |  |  |  |
| Vegetable | ref |  | 0.64 | (-0.19, 1.47) |  | 1.29 | (-0.05, 2.63) |
| Jarred Vegetable | ref |  | n.a. | n.a. |  | -1.02* | (-1.82, -0.22) |
| Fresh Vegetable | ref |  | n.a. | n.a. |  | 1.27* | (0.44, 2.09) |
| **LLDS without meat and fish, SSB, or dairy component** | | | | | | | |
| Dairy | ref |  | 0.20 | (-0.82, 1.22) |  | -0.65 | (-1.72, 0.42) |
| Meat and fish | ref |  | -0.15 | (-1.13, 0.83) |  | -1.43 | (-3.02, 0.16) |
| SSB | ref |  | 0.86 | (-0.43, 2.16) |  | -0.67 | (-1.94, 0.60) |
| **DASH without fruit component** | |  |  |  |  |  |  |
| Fruit | ref |  | 0.52 | (-0.13, 1.18) |  | 0.30 | (-0.31, 0.90) |
| Jarred Fruit | ref |  | n.a. | n.a. |  | -0.92* | (-1.46, -0.39) |
| Fresh Fruit | ref |  | n.a. | n.a. |  | 0.95* | (0.42, 1.48) |
| **DASH without vegetable component** | |  |  |  |  |  |  |
| Vegetable | ref |  | 0.42 | (-0.15, 1.00) |  | 1.31* | (0.39, 2.24) |
| Jarred Vegetable | ref |  | n.a. | n.a. |  | -0.89* | (-1.43, -0.34) |
| Fresh Vegetable | ref |  | n.a. | n.a. |  | 1.00* | (0.43, 1.56) |
| **DASH without meat and fish, SSB, or dairy component** | | | | | | | |
| Dairy | ref |  | 0.29 | (-0.42, 0.99) |  | -0.88 | (-1.63, -0.14) |
| Meat and fish | ref |  | -0.17 | (-0.87, 0.53) |  | -0.02 | (-1.15, 1.12) |
| SSB | ref |  | 0.32 | (-0.58, 1.22) |  | -0.32 | (-1.21, 0.56) |
| **MDS without fruit component** | |  |  |  |  |  |  |
| Fruit | ref |  | 0.13 | (-0.10, 0.36) |  | 0.08 | (-0.13, 0.30) |
| Jarred Fruit | ref |  | n.a. | n.a. |  | -0.02 | (-0.21, 0.17) |
| Fresh Fruit | ref |  | n.a. | n.a. |  | 0.05 | (-0.14, 0.24) |
| **MDS without vegetable component** | |  |  |  |  |  |  |
| Vegetable | ref |  | 0.16 | (-0.03, 0.35) |  | 0.20 | (-0.11, 0.51) |
| Jarred Vegetable | ref |  | n.a. | n.a. |  | -0.12 | (-0.31, 0.06) |
| Fresh Vegetable | ref |  | n.a. | n.a. |  | 0.09 | (-0.10, 0.29) |
| **MDS without meat and fish, or dairy component** | | | | | | | |
| Dairy | ref |  | -0.07 | (-0.29, 0.15) |  | -0.26* | (-0.50, -0.03) |
| Meat and fish | ref |  | 0.11 | (-0.10, 0.32) |  | 0.00 | (-0.35, 0.34) |
| **P*<0.05. LLDS: Lifelines diet score; SSB: sugar-sweetened beverage; DASH: dietary approaches to stop hypertension diet score; MDS: Mediterranean diet score. n.a.: not applicable. Data is shown in b (95% CI). Regression coefficients (b) represent the mean difference in diet quality score compared to the reference category.  For bread, “Mainly white” temporal pattern was considered the reference category. For other food groups, “Low intake” patterns were considered the reference. Model was adjusted for children’s age and sex, maternal education, mother smoked during pregnancy, and the presence of siblings at birth. | | | | | | | |

Fig. S3 Odds for adherence to dietary intake temporal patterns of fruit and vegetables for paternal and family characteristics at baseline of the GECKO cohort


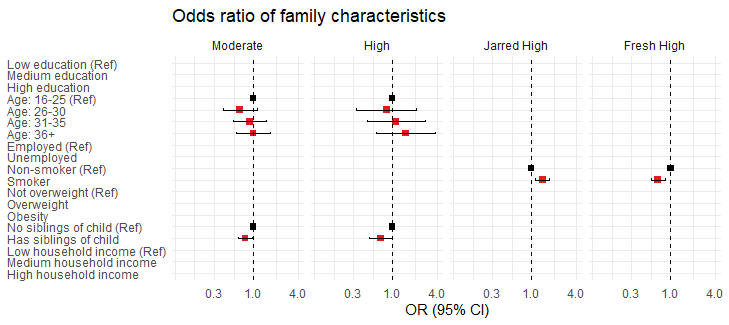

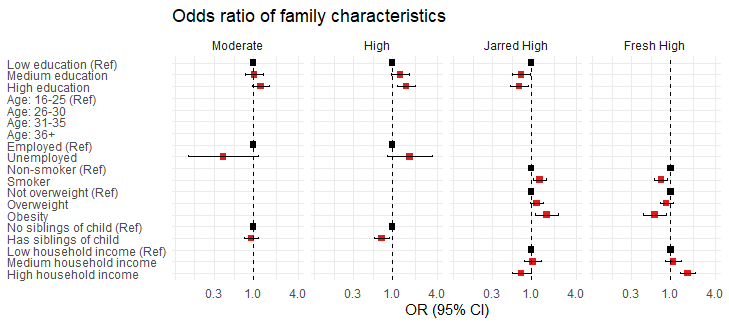


**A Fruit intake**

**B Vegetables intake**

Moderate High Jarred High Fresh High

Reference

Estimate

To enhance clarity, only variables retained in the final model based on the selection criteria are presented in the figures. “Low intake” temporal patterns were considered the reference group.

Fig. S4 Odds for adherence to dietary intake temporal patterns of other food groups for paternal and family characteristics at baseline of the GECKO cohort


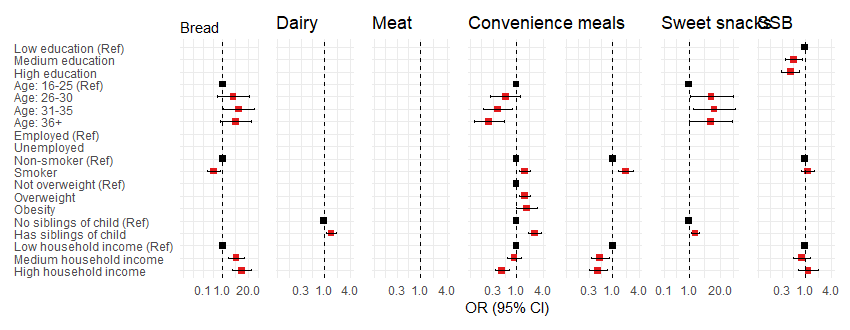

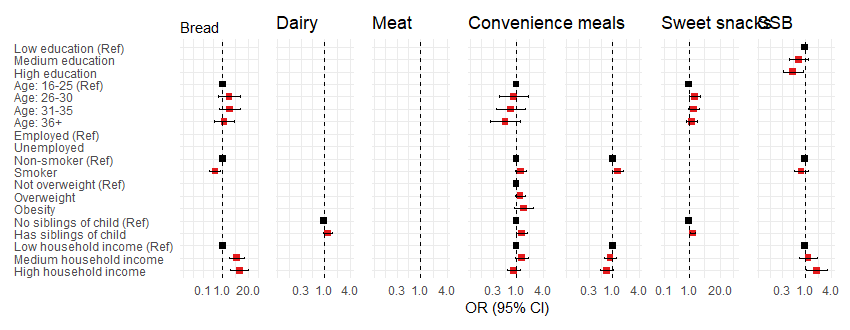


Bread Dairy Meat Convenience meals Savory snacks Sweet snacks SSB

**A Moderate intake**

**B High intake**

Reference

Estimate

To enhance clarity, only variables retained in the final model based on the selection criteria are presented in the figures. For bread, “Mainly white” temporal pattern was considered the reference category. For other food groups, “Low intake” temporal patterns were considered the reference category.
